# Diagnosis of Chronic Kidney Disease Using Retinal Imaging and Urine Dipstick Data: Multimodal Deep Learning Approach

**DOI:** 10.2196/55825

**Published:** 2025-02-07

**Authors:** Youngmin Bhak, Yu Ho Lee, Joonhyung Kim, Kiwon Lee, Daehwan Lee, Eun Chan Jang, Eunjeong Jang, Christopher Seungkyu Lee, Eun Seok Kang, Sehee Park, Hyun Wook Han, Sang Min Nam

**Affiliations:** 1 Korean Genomics Center (KOGIC), Ulsan National Institute of Science and Technology (UNIST), Ulsan, Republic of Korea; 2 Department of Biomedical Engineering, College of Information and Biotechnology, Ulsan National Institute of Science and Technology (UNIST), Ulsan, Republic of Korea; 3 Division of Nephrology, Department of Internal Medicine, CHA Bundang Medical Center, CHA University, Gyeonggi-do, Republic of Korea; 4 Department of Ophthalmology, CHA Bundang Medical Center, CHA University, Gyeonggi-do, Republic of Korea; 5 Spidercore Inc, Daejeon, Republic of Korea; 6 Department of Biomedical Informatics, School of Medicine, CHA University, 335 Pangyo-ro, Seongnam, Republic of Korea, 82 31-881-7964, 82 31-881-7069; 7 Institute for Biomedical Informatics, School of Medicine, CHA University, Seongnam, Republic of Korea; 8 Department of Ophthalmology, Institute of Vision Research, Severance Hospital, Yonsei University College of Medicine, Seoul, Republic of Korea; 9 Division of Endocrinology and Metabolism, Department of Internal Medicine, Yonsei University College of Medicine, Seoul, Republic of Korea; 10 Department of ICT Safety, Graduate School of Chung-Ang University, Seoul, Republic of Korea; 11 IAEC Medical Service, Seoul, Republic of Korea; 12 Institute of Basic Medical Sciences, School of Medicine, CHA University, Gyeonggi-do, Republic of Korea; 13 Healthcare Big Data Center, CHA Bundang Medical Center, Gyeonggi-do, Republic of Korea; 14 Daechi Yonsei Eye Clinic, Seoul, Republic of Korea

**Keywords:** multimodal deep learning, chronic kidney disease, fundus image, saliency map, urine dipstick

## Abstract

**Background:**

Chronic kidney disease (CKD) is a prevalent condition with significant global health implications. Early detection and management are critical to prevent disease progression and complications. Deep learning (DL) models using retinal images have emerged as potential noninvasive screening tools for CKD, though their performance may be limited, especially in identifying individuals with proteinuria and in specific subgroups.

**Objective:**

We aim to evaluate the efficacy of integrating retinal images and urine dipstick data into DL models for enhanced CKD diagnosis.

**Methods:**

The 3 models were developed and validated: eGFR-RIDL (estimated glomerular filtration rate–retinal image deep learning), eGFR-UDLR (logistic regression using urine dipstick data), and eGFR-MMDL (multimodal deep learning combining retinal images and urine dipstick data). All models were trained to predict an eGFR<60 mL/min/1.73 m², a key indicator of CKD, calculated using the 2009 CKD-EPI (Chronic Kidney Disease Epidemiology Collaboration) equation. This study used a multicenter dataset of participants aged 20‐79 years, including a development set (65,082 people) and an external validation set (58,284 people). Wide Residual Networks were used for DL, and saliency maps were used to visualize model attention. Sensitivity analyses assessed the impact of numerical variables.

**Results:**

eGFR-MMDL outperformed eGFR-RIDL in both the test and external validation sets, with area under the curves of 0.94 versus 0.90 and 0.88 versus 0.77 (*P*<.001 for both, DeLong test). eGFR-UDLR outperformed eGFR-RIDL and was comparable to eGFR-MMDL, particularly in the external validation. However, in the subgroup analysis, eGFR-MMDL showed improvement across all subgroups, while eGFR-UDLR demonstrated no such gains. This suggested that the enhanced performance of eGFR-MMDL was not due to urine data alone, but rather from the synergistic integration of both retinal images and urine data. The eGFR-MMDL model demonstrated the best performance in individuals younger than 65 years or those with proteinuria. Age and proteinuria were identified as critical factors influencing model performance. Saliency maps indicated that urine data and retinal images provide complementary information, with urine offering insights into retinal abnormalities and retinal images, particularly the arcade vessels, being key for predicting kidney function.

**Conclusions:**

The MMDL model integrating retinal images and urine dipstick data show significant promise for noninvasive CKD screening, outperforming the retinal image–only model. However, routine blood tests are still recommended for individuals aged 65 years and older due to the model’s limited performance in this age group.

## Introduction

Chronic kidney disease (CKD) is a pervasive and potentially irreversible condition that afflicts more than 10% of the global population [1,2]. It is diagnosed based on the presence of decreased glomerular filtration rate (GFR) or markers of kidney damage, such as proteinuria, persisting for over 3 months [3]. In clinical practice, estimated glomerular filtration rate (eGFR) is widely used and typically calculated based on demographic factors and serum creatinine levels. A drop in eGFR below 60 mL/min/1.73 m^2^ significantly elevates the risk of cardiovascular diseases, mortality, and progression to end-stage kidney disease [4-6]. Proteinuria, indicative of kidney damage, further compounds these risks and affects cardiovascular outcomes and patient survival [6,7]. Efficient CKD screening is critical, as patients are often asymptomatic until the disease reaches advanced stages [8].

Remarkably, the eye and kidney share developmental, structural, physiological, and pathological similarities, hinting at a potential link between ocular and renal diseases [9]. Notably, both organs are highly vascularized and susceptible to conditions affecting the vascular system, such as aging, diabetes, and hypertension [10]. Evidence suggests that individuals displaying retinal microvascular signs, including retinopathy, arteriolar narrowing, and venular dilatation, exhibit an increased predisposition for CKD, and vice versa [1,11-14]. Fundus imaging allows for the convenient assessment of the retinal microvasculature, making it a potential screening modality for incident CKD. However, conventional fundus photography analysis has limitations in predicting CKD incidence or progression due to population heterogeneity and sensitivity issues [1].

To address these limitations, the integration of artificial intelligence, particularly deep learning (DL), with retinal imaging has emerged as a promising approach. Recent studies using DL models (convolutional neural networks, cCondenseNet, or ResNet-50) have demonstrated moderate to good performance in identifying individuals with decreased eGFR or CKD based on retinal photographs [15,16]. Nevertheless, the performance diminishes when detecting isolated proteinuria compared with overall CKD [16].

Convenient and cost-effective proteinuria assessment can be accomplished using a simple urine dipstick test [3]. Combining a retinal image deep learning (RIDL) model for eGFR decline identification with urinalysis for proteinuria detection would eliminate the need for invasive blood tests, thereby significantly improving convenience and practicality. In addition, XGBoost-based machine learning models, including 7 features (age, sex, and 5 urine dipstick measurements: protein, blood, glucose, pH, and specific gravity), successfully detect eGFR decline, especially among nondiabetic individuals younger than the age of 65 years [17].

In this study, we aimed to develop a multimodal deep learning (MMDL) model using 2 noninvasive data sources: retinal images and urine dipstick tests. We anticipate that this integration will enhance the RIDL model performance. The integration of fundus images with other data modalities in DL classifiers has been shown to improve diagnostic accuracy in some cases. For instance, a study demonstrated that combining retinal fundus images with participant metadata (such as race, age, sex, and blood pressure) enhanced the accuracy of anemia detection using DL, indicating that both metadata and fundus images contributed significantly to the prediction [18]. However, in the context of CKD detection, other studies have reported that adding clinical data to fundus image–based models does not necessarily improve performance. Further, one study used a DL algorithm for CKD detection that combined fundus photos with risk factors such as age, sex, ethnicity, diabetes, and hypertension, while another study combined fundus photos with clinical metadata such as age, sex, height, weight, BMI, and blood pressure [15,16]. In both cases, the additional data do not enhance the performance compared to models using fundus images alone.

The synergy between retinal images and urine data was assessed by comparing the performance of a urine-only model and an MMDL model with that of an RIDL model. Additionally, we comprehensively evaluated the model performance across diverse subgroups defined by well-established risk factors, such as age, diabetes, and hypertension [1,19-22]. Identifying data subgroups plays a critical role in model testing and assessing model fairness, as these subgroups can exhibit distinct or unexpected behaviors compared with the overall dataset [23]. Previous studies have explored DL and machine learning model performance within subgroups with varying outcomes. Some studies on DL models observed improved performance in patients with diabetes or high hemoglobin A_1c_, whereas others found consistent performance across diabetes and hypertension subgroups [15,24]. However, no assessment has been conducted for eGFR<60 mL/min/1.73 m^2^, a critical CKD diagnostic threshold [3]. The machine learning model, assessing eGFR<60 mL/min/1.73 m² using urine dipstick tests, exhibited compromised performance in diabetes, especially without proteinuria, but performed well in hypertension, especially in those younger than 65 years of age [17]. Therefore, our study systematically evaluated the model performance in 4 subgroups, including proteinuria, age ≥65 years, diabetes, and hypertension, to provide a comprehensive understanding of its capabilities and limitations in real-world scenarios.

## Methods

### Preparation of Study Data

**Figure 1.**
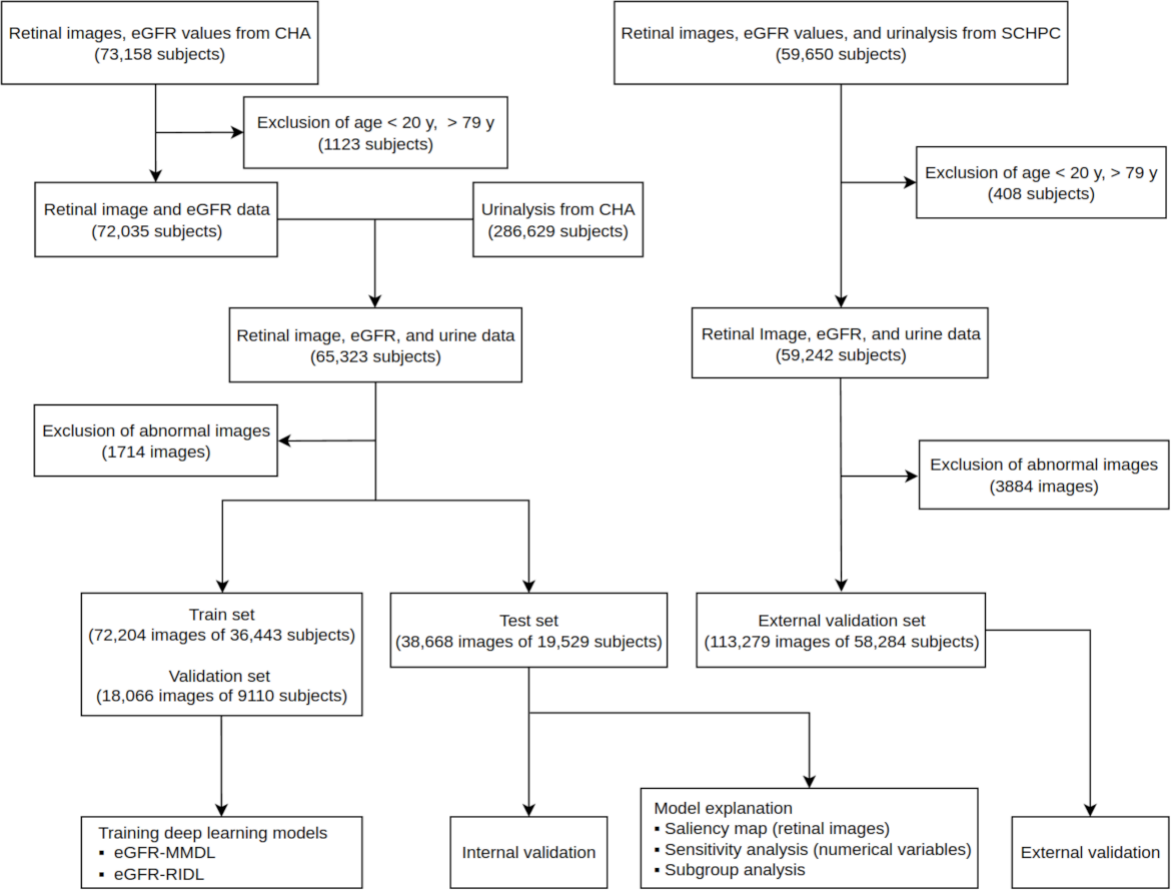
Study flowchart from data preparation to deep learning modeling and validation. Images with abnormal brightness or haziness were excluded. CHA: CHA Bundang Medical Center; eGFR: estimated glomerular filtration rate; eGFR-MMDL: multimodal deep learning model for estimated glomerular filtration rate<60 mL/min/1.73 m²; eGFR-RIDL: retinal image deep learning model for estimated glomerular filtration rate<60 mL/min/1.73 m²; SCHPC: Severance Checkup Health Promotion Center.

Retinal images, serum creatinine levels, urine dipstick results, and demographic data (age, sex, diabetes, and hypertension) were collected from multiple departments at CHA Bundang Medical Center over 12 years (2008‐2019; Figure 1). External validation involved using identical datasets from the Severance Checkup Health Promotion Center (SCHPC) for 8 years (2013‐2020).

The people aged 20‐79 years were selected without duplication. The age range for adulthood was defined as 20‐79 years, with outliers older than 79 years removed using the IQR method. Macula-centered retinal images were taken from CHA Bundang Medical Center using 1 of 2 cameras (TRC-NW8, Topcon; nonmyd 7, Kowa) at the health promotion center or endocrinology department without pupil dilation, or using VX-10i (Kowa) at the ophthalmology department with or without pupil dilation. The same style of retinal images was acquired from SCHPC using different camera models without pupil dilation (CT-80A, Topcon; AFC-210). Cases with unilateral retinal images were not collected to create ensemble models using bilateral retinal images. Serum creatinine and urine dipstick tests were performed on the same day, and retinal images were selected within 35 days before or after these tests. eGFR was calculated using a 2009 CKD-EPI (Chronic Kidney Disease Epidemiology Collaboration) formula based on serum creatinine levels [25].

In Supplementary Methods (Multimedia Appendix 1), we outlined the criteria for diabetes and hypertension, shared details about the urine analyzers used in the model, explained the scoring method for urine dipstick tests, and provided comprehensive information on image preprocessing, including algorithms for excluding abnormal images based on brightness and haziness.

### Ethical Considerations

Ethical approval was granted by the institutional review boards of the Bundang CHA Medical Center (2019-01-032) and Severance Hospital (4-2020-0231), and written informed consent was not required as the data were anonymized.

### Model Training

This study aimed to predict eGFR<60 mL/min/1.73 m² using retinal images and urine dipstick tests. The development dataset was divided into train, validation, and test sets (Figure 1). Further, 2 Wide Residual Network (WRN) models were built: eGFR-RIDL and eGFR-MMDL [26].

**Figure 2.**
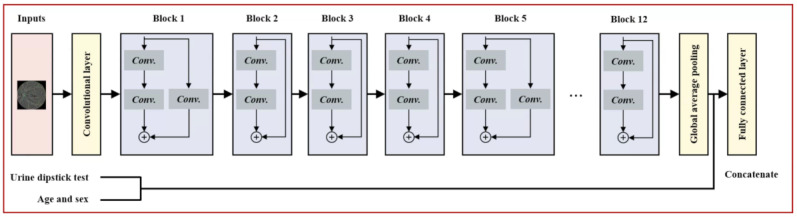
Wide Residual Network architecture for detecting kidney function decline using retinal images. For the multimodal model, the urine dipstick measurements, age, and sex were concatenated with the feature vector from the image. Conv: convolutional layer.

Macula-centered retinal images were processed through a WRN-28-4 [26] and initialized with He initialization (Figure 2). Random left- and right-flip augmentation of the image was performed. In the case of eGFR-RIDL, the 256-dimensional feature vector from the convolutional layers was processed through a fully connected layer with a sigmoid activation function. Conversely, for eGFR-MMDL, the 256D feature vector from the convolutional layers analyzing retinal images was concatenated with a 12D vector representing age, sex, and urinalysis measurements using a joint fusion approach. This combined vector was then fed into a fully connected layer with a sigmoid activation function (Figure 2) [27]. Standardization was performed by removing the mean and scaling all numerical variables, except sex, before concatenation.

The binary cross-entropy function was used to calculate the loss values, and the model parameters were updated using the gradient descent method with the AdamW optimizer. To overcome the issue of imbalanced data, where the eGFR<60 mL/min/1.73 m² class was outnumbered by the eGFR>60 mL/min/1.73 m² class, the loss function was scaled by a “sample_weight” coefficient, which was calculated as the ratio of cases with eGFR>60 mL/min/1.73 m² to those with eGFR<60 mL/min/1.73 m². Model training was monitored using plots of losses and area under the curve (AUC) from the training and validation datasets to avoid overfitting, and the best epoch was identified. Our model algorithm is deployed on GitHub [28] in a runnable form, which takes in retinal images and urine analysis data.

### Development of Urine Dipstick Logistic Regression Model

A logistic regression model was generated solely with numerical variables, including urine dipstick measurements, age, and sex, for comparison with the eGFR-RIDL model. Standardized numerical values were used for training and validation across both test and external validation datasets. When building the logistic regression model, we used the class_weight=“balanced” option to adjust for class imbalance, ensuring that each class was appropriately weighted in the analysis.

### Validation and Subgroup Analysis of the Model Performance

The probabilities from both eyes were averaged to calculate the probability of eGFR<60 mL/min/1.73 m². If only 1 eye image remained after excluding the other eye (Figure 1), the probability of the eye was used. We evaluated the model performance through internal and external validation. Internal validation used the unseen test set from the development data, whereas external validation used the SCHPC dataset. The model’s performance was evaluated using AUC comparisons against the AUC of eGFR-RIDL. Additionally, the performance across various subgroups was assessed by stratifying the test set based on age, diabetes, hypertension, and proteinuria status (trace or higher urine protein). Statistical comparisons of the AUCs were made using the DeLong test, with Bonferroni correction applied when necessary.

The sensitivity and specificity were calculated by finding the best threshold using the Index of Union method, and a 95% CI was determined using bootstrap resampling. Saliency maps for retinal images and sensitivity analysis of numerical variables were used for model interpretation. Software and calculation specifics are detailed in the Supplementary Methods in Multimedia Appendix 1.

### Statistics

A statistically significant difference between the 2 statistics was determined if the *P* value was below .05, or if there was no overlap between the CIs.

## Results

### Baseline Characteristics of the Participants

We initially screened 73,158 people from CHA and 59,650 from SCHPC, excluding those aged <20 years, >79 years, and those with low-quality images. This resulted in 128,938 images from 65,082 people with CHA and 113,279 images from 58,284 SCHPC people, ensuring that each person had at least 1 eligible image for analysis. Table 1 shows the baseline demographics and laboratory parameters of the CHA and SCHPC datasets. The developmental dataset exhibited a greater proportion of individuals younger than the age of 65 years, as well as a higher prevalence of diabetes, hypertension, and eGFR below 60 mL/min/1.73 m² compared to the external validation dataset. The distribution of measurements for the 7 urine tests (blood, protein, glucose, ketone, urobilinogen, bilirubin, and leucocyte) varied between the developmental and external datasets, as evidenced by the histogram plots provided in Figure S2 in Multimedia Appendix 1.

**Table 1.** Baseline characteristics of study data.

Variables		Development data (CHA)^a^	External validation data (SCHPC)^b^	*P* value
**Age (years), mean (SD; range)**		46 (12; 20-79)	48 (12; 20-79)	<.001^c^
	<65, n^d^ (%)	60,195 (92.5)	53,081 (91.1)	<.001^e^
	≥65, n (%)	4887 (7.5)	5203 (8.9)	
**Sex, n (%)**				<.001^e^
	Female	29,718 (45.7)	27,907 (47.9)	
	Male	35,364 (54.3)	30,377 (52.1)	
**Diabetes, n (%)** ^**f**^				<.001^e^
	Positive	6458 (14.2)	2567 (5)	
	Negative	38,977 (85.8)	49,067 (95)	
**Hypertension, n (%)^f^**				<.001^e^
	Positive	11,172 (18.6)	7503 (14.1)	
	Negative	48,897 (81.4)	45,626 (85.9)	
**eGFR**^**g**^ **(mL/min/1.73 m^2^)**				
	Mean (SD)	100.5 (17)	100.4 (14.6)	.20^c^
	<60, n (%)	1085 (1.7)	492 (0.8)	<.001^e^
	<60 in age <65 y, n (%)	474 (0.8)	235 (0.4)	<.001^e^
	<60 in age ≥65 y, n (%)	611 (12.5)	257 (4.9)	<.001^e^
**Urine tests**				
	Specific gravity, median (IQR; range)	1.02 (0.015; 1.005‐1.03)	1.025 (0.01; 1.005‐1.03)	<.001^h^
	Urine pH, median (IQR; range)	6 (1; 5‐9)	5 (0.5; 5‐9)	<.001^h^
	Blood, median (IQR; range)	0 (0; 0‐4)	0 (0; 0‐4)	<.001^h^
	Protein, median (IQR; range)	0 (0; 0‐5)	0 (0; 0‐5)	<.001^h^
	Glucose, median (IQR; range)	0 (0; 0‐5)	0 (0; 0‐5)	<.001^h^
	Ketone, median (IQR; range)	0 (0; 0‐5)	0 (0; 0‐4)	<.001^h^
	Urobilinogen, median (IQR; range)	0 (0; 0‐4)	0 (0; 0‐4)	<.001^h^
	Bilirubin, median (IQR; range)	0 (0; 0‐4)	0 (0; 0‐4)	<.001^h^
	Leucocyte, median (IQR; range)	0 (0; 0‐4)	0 (0; 0‐4)	<.001^h^
	Nitrite, n (%)	570 (0.9)	263 (0.5)	<.001^c^

a CHA: Bundang CHA Medical Center.

b SCHPC: Severance Checkup Health Promotion Center.

c Independent 2-sample 2-tailed *t* test.

d n: number of images.

e Chi-square test.

f Unknown cases were excluded.

g eGFR: estimated glomerular filtration rate.

h Wilcoxon rank-sum test.

### Performance Difference Between Unimodal and Multimodal Models

**Figure 3.**
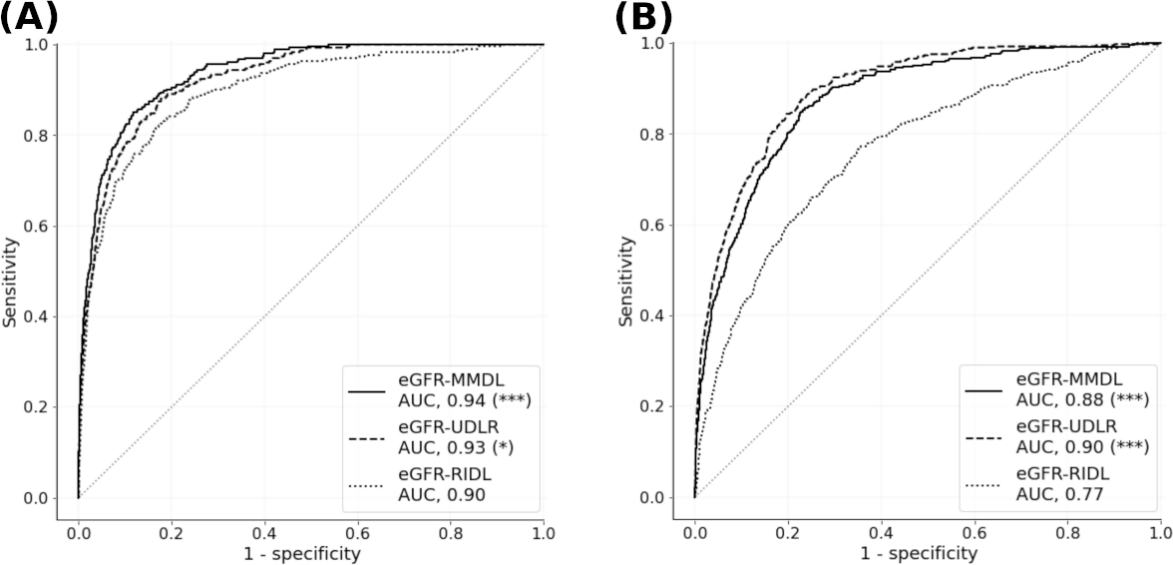
ROC curves and AUC of low-eGFR detection models. The AUCs of the eGFR-MMDL and eGFR-UDLR models were compared with the AUC of eGFR-RIDL in (**A**) the test set and (**B**) the external validation set (DeLong test with Bonferroni correction). *P=.002; ***P<.001. AUC: area under the curve; eGFR: estimated glomerular filtration rate; MMDL: multimodal deep learning model; RIDL: retinal image deep learning model; ROC: receiver operating characteristic; UDLR: urine dipstick logistic regression.

**Figure 4.**
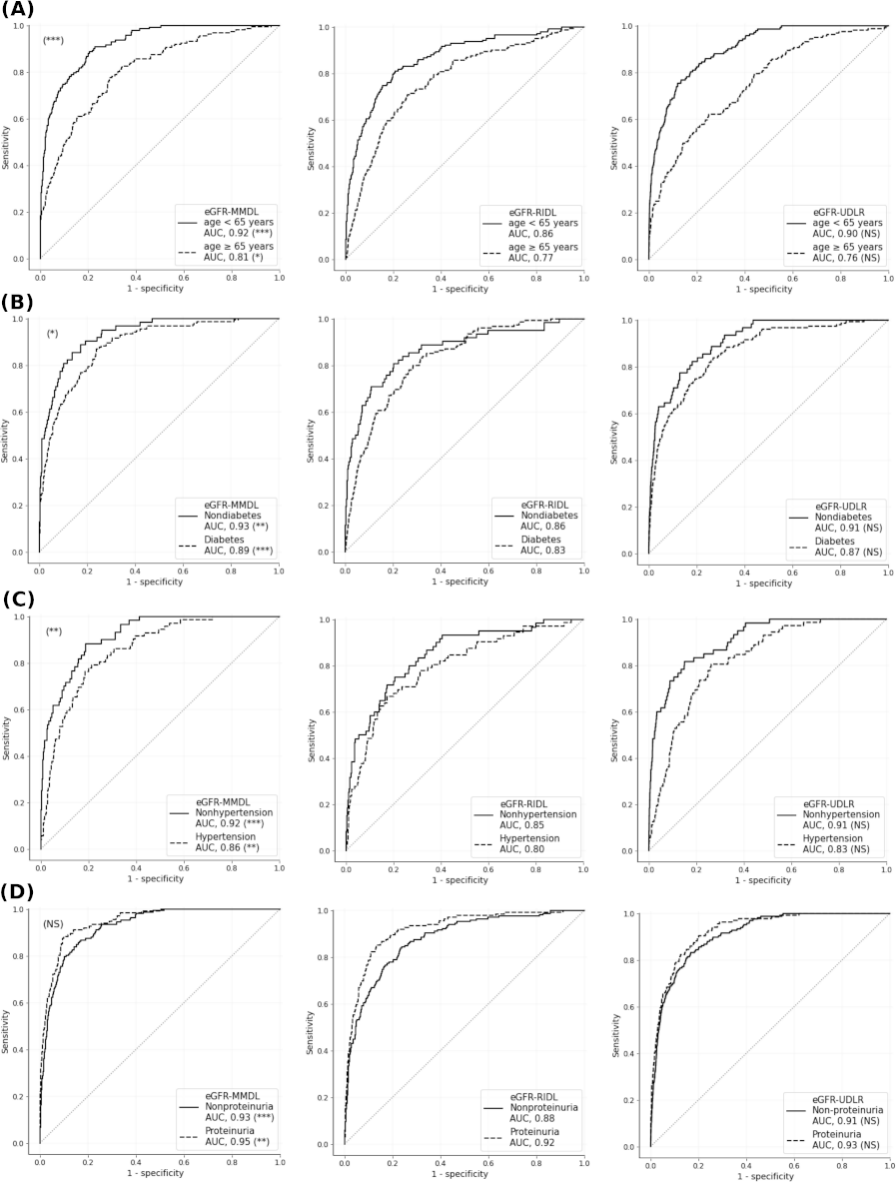
ROC curves and AUC of low-eGFR detection models in subgroups of the test set. (**A**) Age <65 years (solid line) and ≥65 years (dotted line); (**B**) nondiabetes (solid line) and diabetes (dotted line); (**C**) no hypertension (solid line) and hypertension (dotted line); and (**D**) no proteinuria (solid line) and proteinuria (dotted line). The AUCs of the eGFR-MMDL and eGFR-UDLR models are compared with the AUC of the eGFR-RIDL (DeLong test with Bonferroni correction; *P* value indicators next to the AUC values); *P=.013, **P=.001, ***P<.001; NS: not significant. *P* value indicators are added to the upper left corner of the eGFR-MMDL model curve to denote significant differences in AUCs between the 2 subgroups (DeLong test); *P=.011, **P=.001, ***P<.001; NS: not significant. AUC: area under the curve; eGFR: estimated glomerular filtration rate; MMDL: multimodal deep learning model; RIDL: retinal image deep learning model; ROC: receiver operating characteristic; UDLR: urine dipstick logistic regression.

Despite the inclusion of numerical variables in the retinal image data, the training curves revealed a faster learning rate without overfitting in the eGFR-MMDL model than in the eGFR-RIDL model (Figure S3 in Multimedia Appendix 1). Figure 3 highlights the superior performance of the eGFR-MMDL model over the eGFR-RIDL model on internal and external datasets. Moreover, the eGFR-MMDL model demonstrated a notable increase in AUC across all subgroups compared with the eGFR-RIDL model (Figure 4).

In addition, the eGFR-UDLR (urine dipstick logistic regression) performed better than the eGFR-RIDL and was comparable to the eGFR-MMDL model, especially in external validation (Figure 3). However, no significant improvement was observed within subgroups in internal validation (Figure 4).

Age influenced the performance of the eGFR-MMDL model. For the overall population, the model performed better in individuals without diabetes or hypertension compared to their respective control groups, while proteinuria had no impact on performance (Figure 4B, 4C, and 4D). When stratified by the age of 65 years, no significant differences in AUC were observed between the nondiabetes and diabetes groups or the nonhypertension and hypertension groups (Figure S4 in Multimedia Appendix 1). However, the AUC increased in the presence of proteinuria (Figure S4 in Multimedia Appendix 1). These changes appeared to be driven by higher model performance in younger individuals without diabetes, hypertension, or proteinuria (Figure 4A).

Table 2 presents the sensitivity and specificity of the eGFR-MMDL model for both the entire study population and its subgroups. The thresholds for the age group of 65 years and older, as well as for individuals with diabetes and proteinuria, were significantly higher than the overall threshold. Table S1 in Multimedia Appendix 1 provides case counts for each subgroup.

**Table 2.** Sensitivity and specificity at the threshold of estimated glomerular filtration rate–multimodal deep learning.The 95% CIs were calculated using bootstrap resampling. The threshold was obtained using the Index of Union method.

	Sensitivity (95% CI)	Specificity (95% CI)	Threshold (95% CI)
All	0.86 (0.81‐0.91)	0.88 (0.81‐0.91)	0.53 (0.42‐0.62)
**Age (years)**			
≥65	0.78 (0.7‐0.83)	0.71 (0.66‐0.79)	0.79 (0.77‐0.83)
<65	0.88 (0.78‐0.93)	0.81 (0.77‐0.92)	0.35 (0.3‐0.52)
**Diabetes**			
Yes	0.86 (0.79‐0.9)	0.77 (0.74‐0.84)	0.77 (0.74‐0.82)
No	0.88 (0.79‐0.95)	0.86 (0.8‐0.92)	0.38 (0.3‐0.48)
**Hypertension**			
Yes	0.8 (0.71‐0.88)	0.79 (0.71‐0.84)	0.52 (0.42‐0.58)
No	0.88 (0.78‐0.93)	0.82 (0.81‐0.88)	0.32 (0.3‐0.4)
**Proteinuria**			
Yes	0.89 (0.84‐0.94)	0.9 (0.86‐0.92)	0.74 (0.65‐0.78)
No	0.85 (0.78‐0.92)	0.86 (0.77‐0.9)	0.47 (0.33‐0.55)

### Model Explanation Using Saliency Maps and Sensitivity Analysis

**Figure 5.**
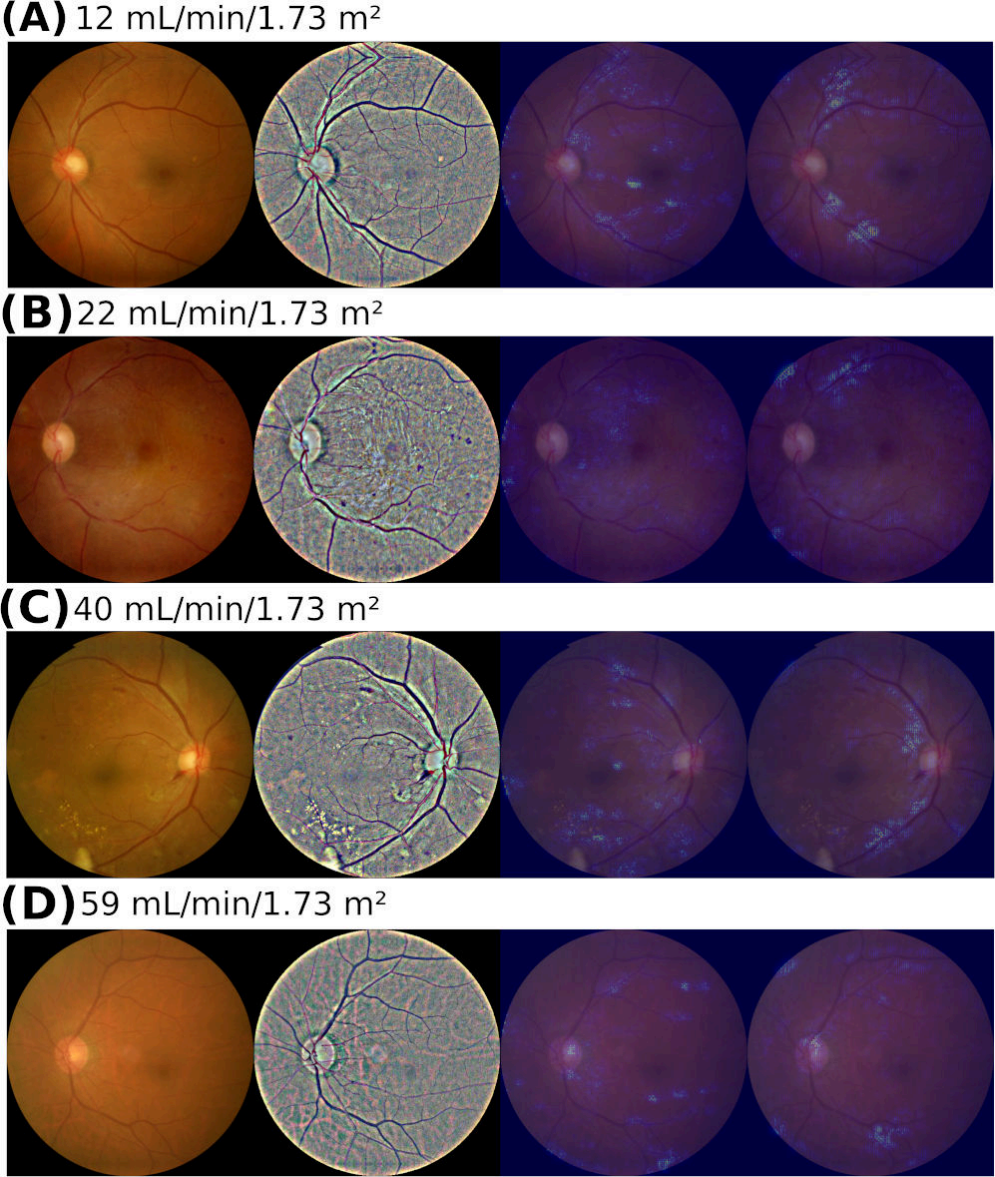
Saliency map results from the eGFR-RIDL and eGFR-MMDL models for eGFR decline detection. (**A**) A nondiabetes male aged 60 years with proteinuria level 3 (2+). (**B**) A male with diabetes and proteinuria level 4 (3+) aged 54 years. (**C**) A male with diabetes and proteinuria level 4 (3+) aged 60 years. (**D**) A male without hypertension or diabetes aged 60 years, with a proteinuria level of 0 (negative). From left to right: original retinal image, retinal image postpreprocessing via CLAHE and color normalization, eGFR-RIDL–generated saliency map, and eGFR-MMDL–generated saliency map. CLAHE: Contrast Limited Adaptive Histogram Equalization; eGFR: estimated glomerular filtration rate; MMDL: multimodal deep learning; RIDL: retinal image deep learning.

To assess the ability of our algorithm to incorporate retinopathy findings in predicting eGFR decline, we examined fundus photographs of patients with diabetic retinopathy (Figures 5B and 5C). The saliency maps from the eGFR-RIDL and eGFR-MMDL models displayed different hot spot distributions. The eGFR-RIDL map highlighted hot spots of retinal abnormalities (hard exudates, retinal hemorrhages, and arteriovenous nicking), small arteries, and veins. Conversely, the eGFR-MMDL map primarily featured hot spots along the central vascular arcade.

To explore the factors used by our algorithm to predict eGFR decline in the absence of definite pathological findings, we analyzed fundus photographs of patients without diabetes (Figures 5A and 5D). Hot spots were observed in the branches of arteries and veins in both the eGFR-RIDL and eGFR-MMDL models, with differing locations between the 2 models.

**Figure 6.**
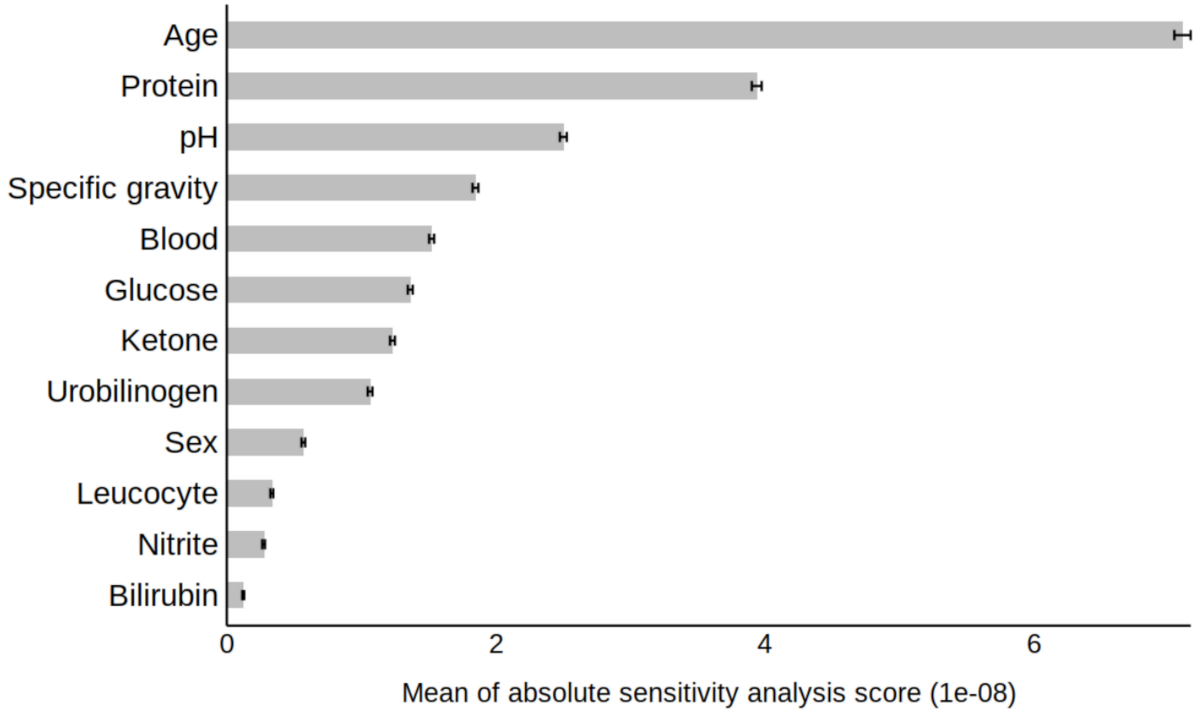
Sensitivity analysis: feature importance of age, sex, and 10 urine measurements in predicting the probability of eGFR decline by eGFR-MMDL using a test set. The sensitivity analysis scores indicate the relative impact of each variable on the predicted probability. Higher sensitivity analysis scores correspond to a greater influence. The error bars on the right end represent 95% CIs. 1e-08: 0.00000001; eGFR: estimated glomerular filtration rate; MMDL: multimodal deep learning.

In the sensitivity analysis of eGFR-MMDL, age and urine protein level were the most influential factors among the numerical variables, followed by urine pH, specific gravity, hematuria, and glycosuria (Figure 6).

## Discussion

### Principal Findings

This study found that the eGFR-MMDL model, which integrates retinal imaging with urine dipstick data, achieved higher diagnostic performance for CKD than the retinal image-only eGFR-RIDL model. The eGFR-MMDL model demonstrated superior accuracy across subgroups, particularly in younger individuals and those without proteinuria. Sensitivity analysis identified age and proteinuria as key factors from urine dipstick data, while saliency maps indicated that retinal vessels provided additional diagnostic information unavailable from urine dipstick data, demonstrating how each modality contributed unique and valuable insights for CKD detection.

### Addressing Variability in Fundus Images for Robust DL Models

In practice, fundus images vary in size, quality, format, and color across different devices [29]. This variability poses a challenge for the creation of effective DL models. Training a model based solely on data from specific devices can limit its compatibility with others. To address this, we used 3 camera models from 2 companies for training and evaluated the model using 2 different nontraining camera models. We standardized the images across devices through preprocessing techniques, including circular border adjustment, boundary removal via cropping, Contrast Limited Adaptive Histogram Equalization–based quality enhancement, and color normalization (Supplementary Methods in Multimedia Appendix 1). Nevertheless, the effectiveness of the model diminished during the external validation, which can be partially attributed to the diversity observed in the retinal images. Notably, the eGFR-UDLR model, leveraging numerical variables, exhibited enhanced generalization compared with the eGFR-RIDL model using retinal images (Figure 3B). Therefore, a multimodal approach incorporating urine dipstick tests could partly address the generalization problem of the eGFR-RIDL model.

In addition, when used on images of poor quality, there is a notable decline in performance owing to the disruption of both the structural and statistical properties of neighboring pixels caused by image degradation [30]. Therefore, our workflow involved image quality management and preprocessing for model accuracy and reliability [1]. Abnormal images, defined as those with haziness, extreme brightness, or darkness, were excluded (additional methods in Multimedia Appendix 1). Our approach assesses clarity using haze grading, which combines high-pass filtering and power spectrum integration across spatial frequencies and gauges intricate detail visibility [31]. Unlike previous methods that manually exclude subpar images or use unspecified techniques for DL, our innovation emphasizes practical, real world, and applicable image quality control processes [15,16,24].

### Enhancement of the Model Performance in MMDL

During internal validation, the eGFR-RIDL model achieved an AUC of 0.9 (Figure 3), indicating its statistical comparability with prior models of retinal images. For example, AUCs were observed at 0.826 (95% CI 0.818‐0.833; ResNet18 model) for eGFR<60 mL/min/1.73 m² with diabetes [32], 0.911 (95% CI 0.886‐0.936; cCondenseNet model) for eGFR<60 mL/min/1.73 m² [15], and 0.918 (95% CI 0.905‐0.933; ResNet-50 model) for eGFR<60 or>60 mL/min/1.73 m² with albuminuria [16]. However, the eGFR-MMDL model performed significantly better than the eGFR-RIDL model during the internal and external validations (Figure 3).

The eGFR-UDLR model demonstrated performance comparable to the eGFR-MMDL model (Figure 3), and the superior performance of eGFR-MMDL over eGFR-RIDL can be attributed to the inclusion of urine dipstick data. However, in the subgroup analysis, the eGFR-MMDL model outperformed eGFR-RIDL across all subgroups, while the eGFR-UDLR model showed no significant improvement across subgroups (Figure 4). This suggests that the synergy between the 2 modalities in the eGFR-MMDL model likely resulted from the effective integration of multimodal data through joint fusion (Figure 2) [33]. This method combines feature representations from neural network layers with data from various sources, such as retinal images and urine analysis, thereby enhancing the model performance. Type 2 joint fusion in eGFR-MMDL improves the correlation across modalities, facilitating more efficient shared representation learning [33]. This approach continually updates the feature representations, thereby enhancing its effectiveness [33]. Furthermore, research has consistently emphasized the advantages of multimodal approaches over unimodal approaches [27]. Multimodal models leverage information from different sources and create comprehensive and robust data representations [27]. This overcomes the limitations associated with individual modalities, including noise [27].

The subgroup analysis highlighted the improved performance of eGFR-MMDL, especially in those aged <65 years without proteinuria (Figure 4). This is crucial because this age group often skips check-ups because of perceived good health and lack of symptoms [34]. Detecting CKD in young individuals is vital, as it can be linked to rare conditions, such as glomerulopathy, where treatments greatly impact outcomes [35]. Furthermore, the good performance of the eGFR-MMDL model in individuals without proteinuria indicated its ability to identify nonproteinuria CKD cases, which usually necessitates invasive blood tests for screening purposes. Thus, eGFR-MMDL may replace blood tests with retinal images and urine dipsticks, offering a convenient, noninvasive screening method for newly diagnosed CKD in young patients.

### Unveiling the Role of Retinal Features and Numerical Variables in Model Prediction

To elucidate these models, we used saliency maps for retinal images and conducted a sensitivity analysis of numerical variables. Kang et al [24] reported that a DL model using saliency maps from retinal images successfully identified common retinal abnormalities, aiding the assessment of renal function. These features, which are routinely used by ophthalmologists for diagnosing retinal diseases, were similarly observed in eGFR-RIDL saliency maps (Figure 5). However, the eGFR-MMDL saliency map notably accentuated arcade vessels over exudation or hemorrhage, which could be attributed to the inclusion of numerical features, such as urine data, age, and sex (Figure 5A). These findings align with those of existing studies that emphasize a strong association between retinal vascular changes and CKD development [36-40]. Joo et al [41] also noted a more pronounced presence of arcade vessels on saliency maps in individuals with higher CKD risk. This suggests that, while urine data provide insights into common retinal abnormalities, retinal images, particularly those focusing on arcade vessels, are pivotal for predicting kidney function. The distinct information yielded by these 2 modalities likely accounted for their complementary roles in the eGFR-MMDL model.

The saliency maps further underscored the significance of the retinal vessel features (Figure 5). Even in cases with healthy retinal images, prominent retinal vessel features indicate their value in predicting renal function (Figures 5A and 5D). These observations align with the findings of Zhang et al [16], emphasizing the relevance of vascular health through saliency maps. However, despite the presence of these features, eGFR-MMDL outperformed eGFR-RIDL (Figure 3), suggesting that retinal vessel features alone may not be sufficient to predict eGFR decline accurately.

In our sensitivity analysis, we assessed the impact of each numerical variable (age, sex, and 10 urine variables) on eGFR<60 mL/min/1.73 m² predictions. The findings on feature importance from the eGFR-MMDL model were consistent with a previous study using XGBoost for eGFR decline prediction, which highlighted urine protein, blood, glucose, pH, specific gravity, and age as important features [17]. In our study, age emerged as the primary contributor, consistent with epidemiological studies, owing to its association with GFR decline [42]. The second most influential factor was urine protein positivity, supported by evidence linking it to kidney function deterioration [43,44]. Urine pH and specific gravity, ranking third and fourth, have been investigated as relevant CKD indicators, with acidic urine pH (5‐5.5) positively correlated with CKD, and low urine specific gravity indicating reduced kidney function [45,46]. Hematuria, the fifth-ranked urine variable, correlates not only with proteinuria but also with CKD risk factors [45,47]. Additionally, glycosuria (urine glucose) may be linked to kidney dysfunction in Fanconi syndrome and diabetes, as indicated in a previous study [45].

### Limitations

First, the performance of eGFR-MMDL in individuals aged ≥65 years did not achieve a satisfactory AUC (Figure 4A), despite the high CKD prevalence in this group (Table 1) [48]. These findings corroborate with those of a previous study, revealing limited predictive capabilities among those aged 65 years and older using urine dipstick-based models [17]. Potential reasons for this underperformance may include issues with the retinal image quality, particularly haziness. However, our analysis revealed no significant difference in eGFR-MMDL performance between the high- and low-haziness groups in individuals aged ≥65 years (Figure S5 in Multimedia Appendix 1).

Furthermore, signs of organ damage, such as retinal abnormalities or proteinuria, may not manifest in older individuals with an eGFR<60 mL/min/1.73 m². This is supported by a higher positive proteinuria ratio in individuals younger than 65 years than in those aged 65 years or older with eGFR<60 mL/min/1.73 m² (263/474, 55.5% vs 243/611, 39.8%, respectively; *P*<.001, chi-square test on the CHA dataset). In people younger than 65 years of age, eGFR<60 was possibly linked to mechanisms causing proteinuria, whereas in those aged 65 years or older, this might be attributed to age-related GFR decline [20]. Given the high prevalence of CKD and the limited performance observed among individuals aged ≥65 years, we recommend conducting routine blood tests for effective CKD screening in this subpopulation.

Second, various medications prescribed for diabetes and hypertension can influence urine dipstick test outcomes, potentially impacting the predictive model accuracy. For example, SGLT2 inhibitors, commonly used in diabetes management, cause glycosuria and may alter urinary specific gravity, while renin-angiotensin-aldosterone inhibitors can decrease proteinuria [49,50]. Unfortunately, due to limited information on specific medication classes, such as renin-angiotensin-aldosterone inhibitors and SGLT2 inhibitors, we were unable to conduct a thorough analysis of their effects on the model performance. Although the eGFR-MMDL model showed robustness to the presence of hypertension or diabetes when age was controlled (Figure S4 in Multimedia Appendix 1), further investigation into medication effects is warranted.

Third, this study’s participants were exclusively Korean, limiting generalizability to diverse ethnic populations. CKD epidemiology and clinical presentations vary across countries, possibly affecting the model performance [51]. Fourth, a gap existed between the dates of retinal imaging and serum or urine tests. However, the absolute difference in dates was small, with a mean of 0.6 (SD 3.7) days.

### Benefits of CKD Screening With Retinal Imaging and Urine Dipstick

The use of noninvasive tests such as retinal imaging and urine dipstick assessments significantly enhances the effectiveness, accessibility, and efficiency of CKD screening, benefiting both patients and health care providers. For patients, these tests reduce discomfort and anxiety, leading to higher compliance with regular screenings and enabling earlier detection of CKD, which is crucial for preventing progression to end-stage kidney disease. This is particularly valuable in resource-limited settings where traditional blood tests may not be readily available [2,52].

For health care providers, these models streamline the screening process by reducing the time and resources needed for blood collection and analysis. The multimodal approach integrates retinal images with urine dipstick data, mirroring the comprehensive assessments made in clinical practice, such as evaluating diabetic or hypertensive retinopathy alongside CKD [27]. This holistic view not only improves diagnostic accuracy but also allows for concurrent evaluation of eye health, providing a more complete picture of the patient’s overall condition.

### Conclusion

The MMDL model, incorporating age, sex, urine measurements, and retinal images, improved the eGFR reduction prediction of an RIDL model. This achievement stemmed from the diverse features of the multiple sources. However, the DL model using retinal images showed limited performance in old-age patients. Our model offers a noninvasive and convenient screening tool for enhancing kidney health in specific populations.

## Supplementary material

10.2196/55825 Multimedia Appendix 1 Figures, tables, and notes.

## Abbreviations

AUC: area under the curve
CKD: chronic kidney disease
CKD-EPI: Chronic Kidney Disease Epidemiology Collaboration
DL: deep learning
eGFR: estimated glomerular filtration rate
GFR: glomerular filtration rate
MMDL: multimodal deep learning
RIDL: retinal image deep learning
SCHPC: Severance Checkup Health Promotion Center
UDLR: urine dipstick logistic regression
WRN: Wide Residual Network
