# Supplementary material for: Diagnosis of Chronic Kidney Disease Using Retinal Imaging and Urine Dipstick Data: Multimodal Deep Learning Approach
Source: JMIR Med Inform. 2025 Feb 7;13:e55825. doi: 10.2196/55825 (PMC11830489; doi:10.2196/55825)
Supplement: Multimedia Appendix 1 [file medinform-v13-e55825-s001.docx]

# Supplementary Figures


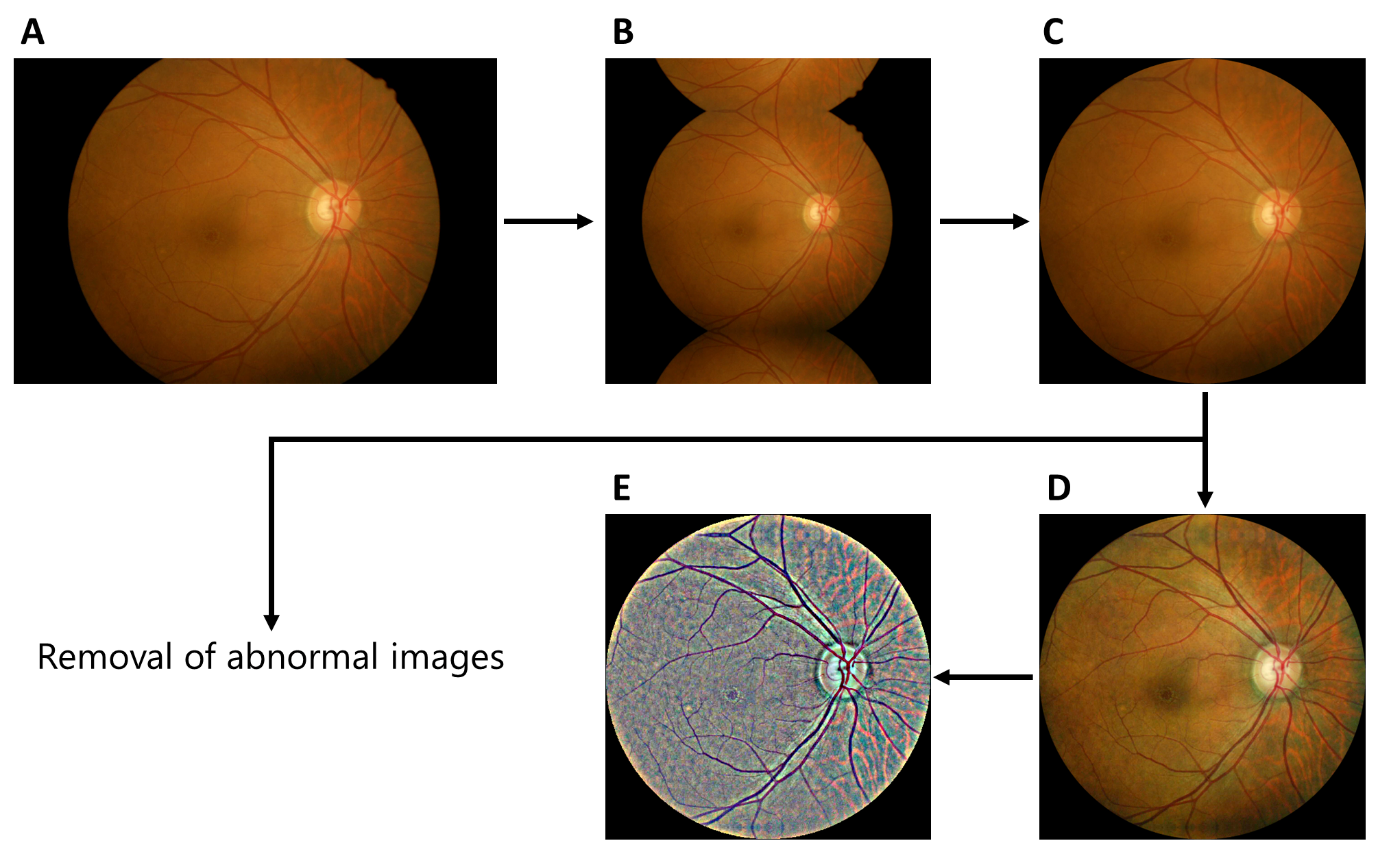


**Figure S1.** Retinal image preprocessing and enhancement. When a retinal image is not fully circle-shaped without the upper and lower borders (A), borders are created (B). The retinal area is cropped, and the circular area is obtained by a circle mask removing boundary effects (C). After excluding images with abnormal brightness or haziness, Contrast Limited Adaptive Histogram Equalization (CLAHE) (D) and color normalization (E) are applied.


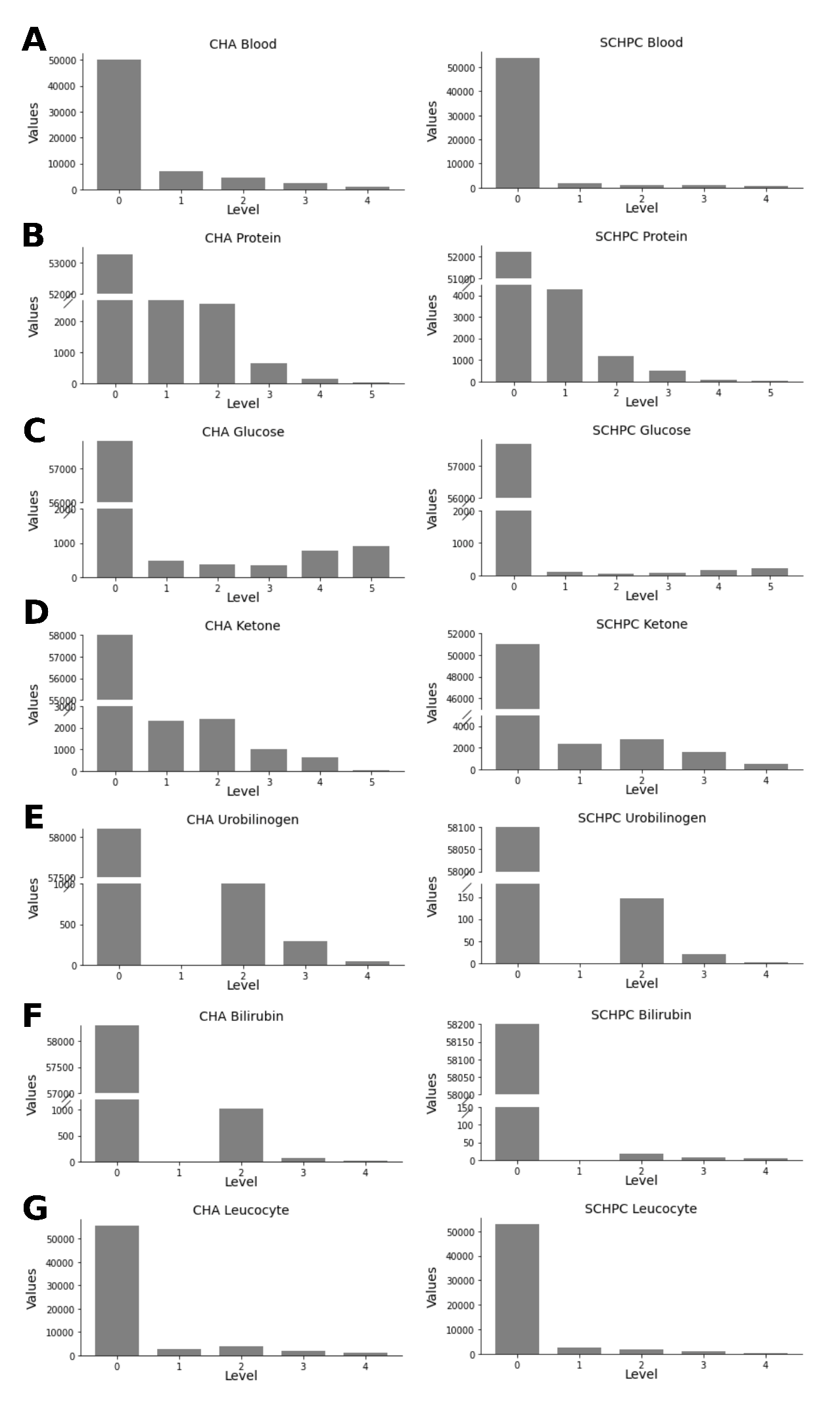


**Figure S2.** Histogram plots displaying the distribution of seven urine measurements within the developmental (CHA) and external validation (SCHPC) datasets. Urobilinogen and bilirubin have zero counts in category one because the values for that category were combined with those of category zero.


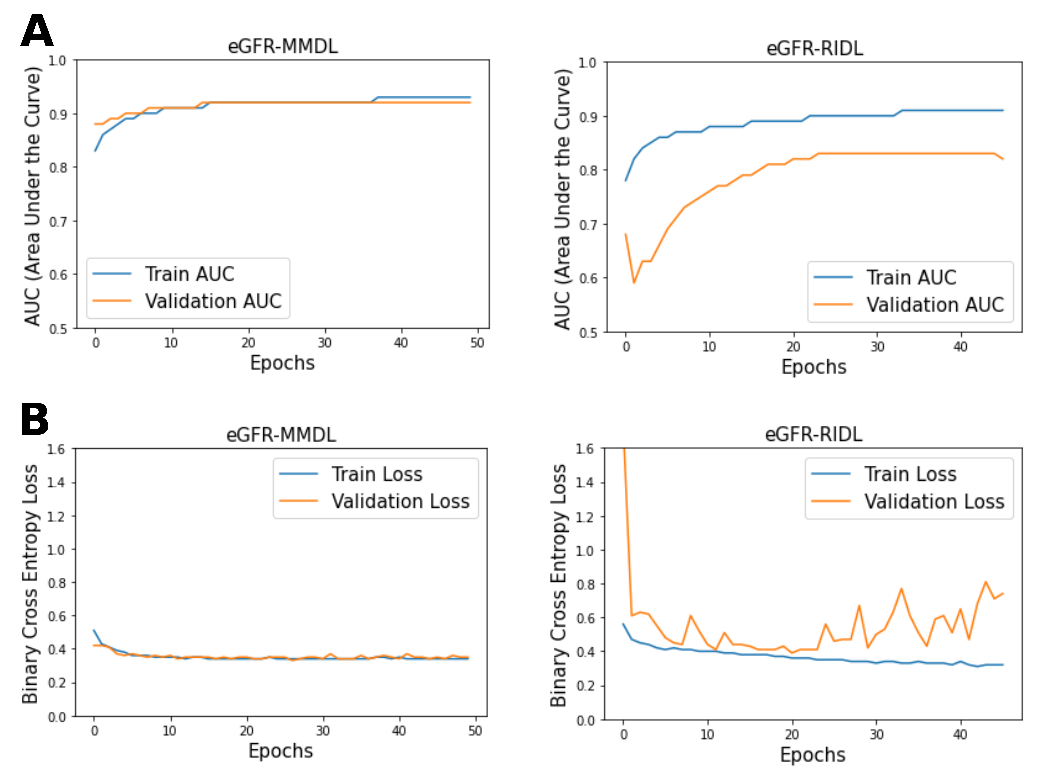


**Figure S3.** Performance (A) and learning (B) curves of eGFR-MMDL and eGFR-RIDL models. eGFR-MMDL model training exhibits less overfitting with a faster learning rate than eGFR-RIDL model training. AUC, areas under the curves; eGFR, estimated glomerular filtration rate; MMDL, multimodal deep learning; RIDL, retinal image deep learning


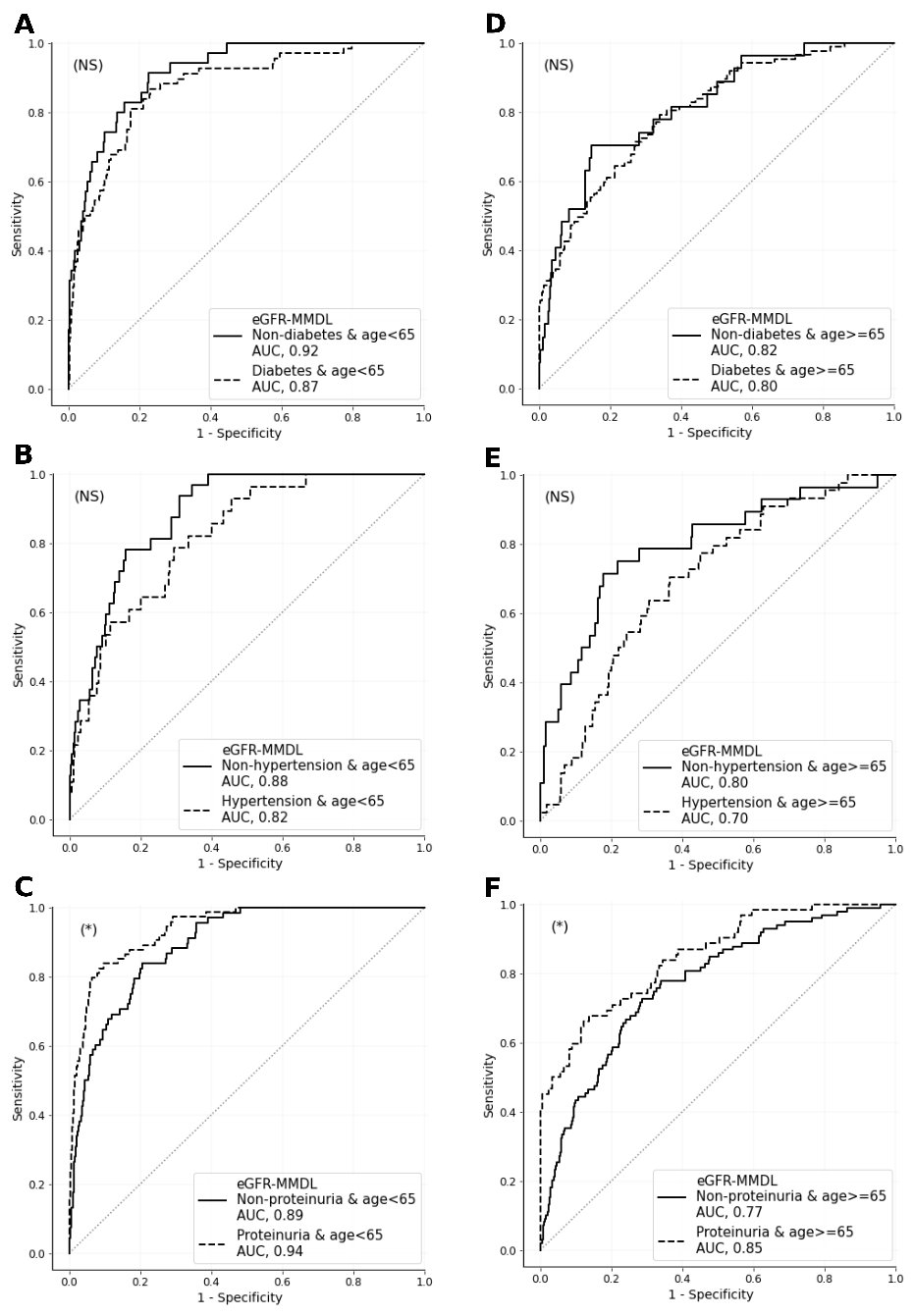


**Figure S4.** Receiver operating characteristic curves and area under the curve (AUC) of eGFR-MMDL for subgroups of age < 65 years (A, B, C) and age ≥ 65 years (D, E, F) from the test set. AUCs are compared for each pair of subgroups using the DeLong test. *, *P*<.05; **, *P*<.01; ***, *P*<.001; NS, not significant. eGFR, estimated glomerular filtration rate; MMDL, multimodal deep learning.


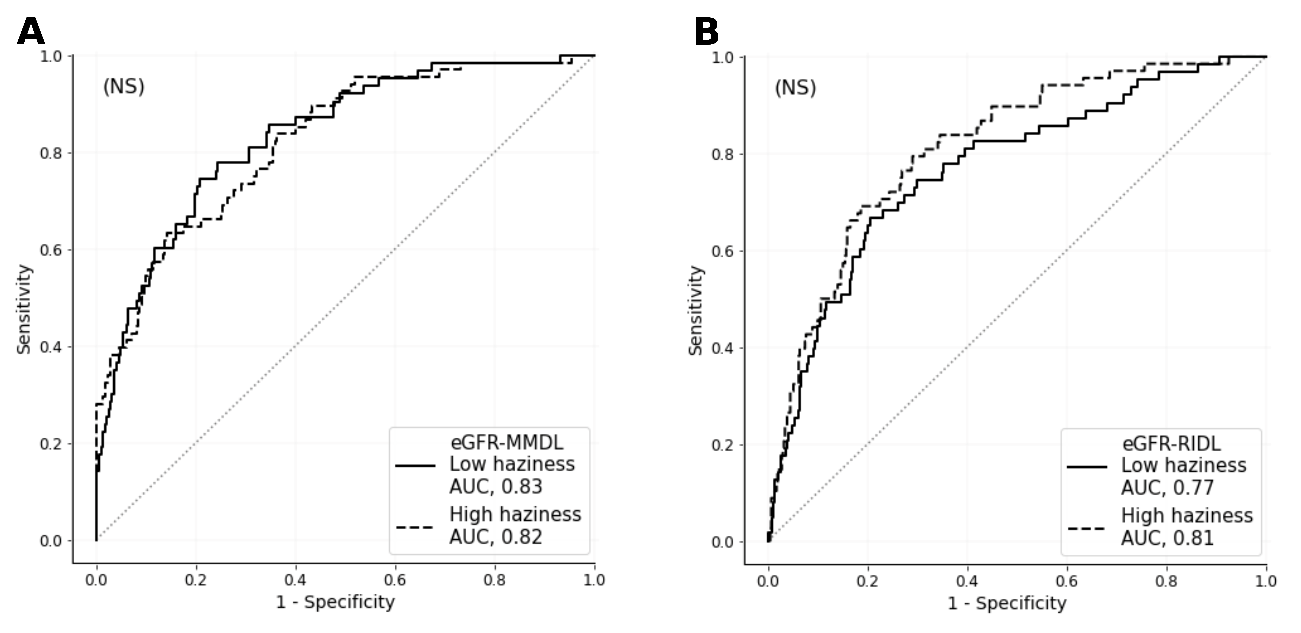


**Figure S5.** Receiver operating characteristic curves and area under the curve (AUC) of eGFR-MMDL (A) and eGFR-RIDL (B) for subgroups of age ≥ 65 years with low or high retinal image haziness from the test set. The Supplementary methods describe the details of the analysis. The AUC of high haziness is compared with that of low haziness for each model (DeLong test). *, *P*<.05; **, *P*<.01; ***, *P*<.001; NS, not significant. eGFR, estimated glomerular filtration rate; MMDL, multimodal deep learning; RIDL, retinal image deep learning.

# Supplementary Table

**Table S1.** Number of cases in each subgroup of the test set (CHA).

| **Subgroups** | **eGFR < 60** | **eGFR ≥ 60** |
| --- | --- | --- |
| Age ≥ 65 years | 161 | 1239 |
| Age < 65 years | 142 | 17,597 |
| Diabetes | 155 | 1722 |
| Non-diabetes | 62 | 11,388 |
| Hypertension | 72 | 3208 |
| Non-hypertension | 60 | 14,396 |
| Proteinuria | 136 | 3322 |
| Non-proteinuria | 167 | 15,514 |

eGFR, mL/min/1.73 m²

# Supplementary Methods

## Criteria of diabetes and hypertension

Diabetes diagnosis relied on medication usage or HbA1c levels exceeding 6.5%, while hypertension was determined by systolic blood pressure ≥ 140 mmHg, diastolic blood pressure ≥ 90 mmHg, or antihypertensive medication use.

## Serum creatinine and urine data information

The urine dipstick test covered ten parameters, each scored as follows: blood, glucose, ketones, and protein were scored from 0 (negative) to 5 (4+), while bilirubin, urobilinogen, and leukocytes were scored from 0 (negative) to 4 (3+). Nitrite was scored as either 0 (negative) or 1 (positive). Negative and trace results for urine bilirubin and urobilinogen were combined at levels of 0. Urobilinogen 4+ at SCHPC was scored as 4 to match the CHA Bundang Medical Center's scale. The pH and specific gravity were reported in 0.5 units from 5 to 9 and 0.005 units from 1.005 to 1.030, respectively.

Urinalysis was conducted using various machines at CHA Bundang Medical Center (URiSCAN® S-300 YD Diagnostics, Yongin, Republic of Korea; URiSCAN® Pro-300 YD Diagnostics, Yongin, Republic of Korea; Clinitek 500 Siemens Healthineers, Erlangen, Germany; AX-4030 Sysmex, Kobe, Japan; UC3500 Sysmex, Kobe, Japan; UC1000 Sysmex, Kobe, Japan) and SCHPC (URiSCAN® Pro II or URiSCAN® PRO, YD Diagnostics, Yongin, Republic of Korea).

## Retinal image preprocessing

The images were classified according to size and assigned a center point (x, y) and a radius r. A retinal circle mask was then created based on these coordinates. If the retinal image was not a complete circle and had missing upper or lower borders, the OpenCV function "copyMakeBorder" was used to fill in these areas using the "BORDER_REFLECT" option (Figure S1B). The contour of the retinal circle was obtained using the "findContours" function of OpenCV, with the options "RETR_EXTERNAL" and "CHAIN_APPROX_SIMPLE". The bounding box surrounding the circle was then determined using the "boundingRect" function of OpenCV and represented by the coordinates (x, y) and (w, h). The image was then cropped based on these bounding box coordinates using the "copyMakeBorder" function in OpenCV. The area surrounding the retinal circle was then made black, and the image was resized to a resolution of 512 × 512 pixels.

The pixel values of the RGB retinal images were standardized by subtracting the mean pixel value from each pixel value and dividing the result by the standard deviation of the pixel values in the image.

## Exclusion of abnormal images

The RGB image was pre-processed and converted into an HSV image. The sum of the V values was then calculated for each quadrant of the image (quadrant-v). Outliers for quadrant-v were determined using the interquartile range (IQR) method, where values falling below Q1 (the lower quartile) - 1.5 IQR or above Q3 (the upper quartile) + 1.5 IQR were considered outliers. If any quadrant-v of an image was identified as an outlier, indicating that the image had areas that were too bright or dark, it was excluded from further analysis.

Then, a scoring algorithm was employed to calculate the haze score for all images, and the upper haze boundary was identified as the point at 99.5% of the haze score distribution [1]. Images with haze scores exceeding the upper limit were excluded from further analysis.

## Retinal image enhancement

The preprocessed images were subjected to Contrast Limited Adaptive Histogram Equalization (CLAHE) using the "createCLAHE" function of OpenCV, with options set to clipLimit = 2.0 and tileGridSize = (8,8) (Figure S1D). The image color was normalized using a previously reported method, where a scale value of 200 was used (Figure S1E) [2].

## Bootstrap confidence intervals and optimal threshold calculation

The bootstrap method was used to estimate the confidence intervals of the model performance metrics [3]. The model outcome was sampled with replacement in a stratified manner 10,000 times, and statistics were computed for each sample. The resulting values were then sorted, and the lower and upper limits of the 95% confidence interval were determined by selecting 2.5% and 97.5% values from the sorted list, respectively.

We determined the optimal threshold by calculating the minimum value of the Index of Union, in combination with the minimum value of the absolute difference between sensitivity and specificity (Index of Union method) [4].

## Saliency map

We utilized a saliency map to gain insight into the important features utilized by the deep learning model to make predictions [5]. To generate the saliency map, we computed the gradient of the model output for the input image. Next, we computed the absolute value of the gradient and obtained the maximum value across the color channels. The resulting values are normalized to obtain the final saliency map. Finally, we blended the saliency map with the input image using equal weights of 0.5.

## Importance of numerical features assessed by sensitivity analysis

The Sensitivity Analysis Score[6] was calculated by subtracting the two predicted probabilities and dividing the result by a small value, 2×eps (epsilon), with eps set to 1e-6. To obtain these two probabilities, we perturbed each numerical variable by adding small positive and negative values (-eps and eps) to the original value and then passed these perturbed values through the neural network model one at a time for all numerical variables in a single sample.

Next, the sensitivity analysis scores were computed for the entire test set of the tabular data. Absolute and average operations were applied to the scores of all the samples in the dataset. The result was depicted in a horizontal bar plot, showing the mean absolute sensitivity analysis scores of the 12 numerical variables. Error bars indicating a 95% confidence interval were displayed at the right end of the bars.

## Impact of retinal image haze intensity on performance in older adults

We aimed to investigate how the intensity of retinal image haze affects the performance of individuals aged ≥ 65 years. To achieve this, we divided the test dataset, consisting entirely of individuals aged 65 years or older, into two groups based on the haze level in their retinal images. We used a median haze score of 0.397, derived from a dataset of 2,912 retinal images of individuals in the same age group, to determine whether an image belonged to the low or high haze group. Of these images, 1,447 were classified as low haze, while the remaining 1,465 were categorized as high haze.

## Software

Python 3.8 programming language[7] and its libraries were used for statistics and calculations, for example, numpy 1.19.5[8] and pandas 1.3.5[9] for data processing; ttest_ind, chi2_contingency, and ranksums functions of Scipy 1.7.3[10] for Table 1; tensorflow-gpu 2.7.0[11] and keras 2.7.0[12] for deep learning; Opencv-python 4.5.5.64[13] functions for image preprocessing and enhancement (Supplementary Methods); roc_curve, aucs, roc_auc_score functions and the LogisticRegression class of scikit-learn 1.0.2[14] for performance evaluation.

Both roc and roc.test functions of pROC R package 1.18.5[15] were used for DeLong test.

The model training was performed on a server with 96 processors, 503GB RAM, two NVIDIA GeForce RTX 2080 Ti GPUs, and CUDA 11.2 and cuDNN 8.4.0 installed.

# References

1. Passaglia CL, Arvaneh T, Greenberg E, Richards D, Madow B. Automated method of grading vitreous haze in patients with uveitis for clinical trials. Transl Vis Sci Technol 2018 Mar;7(2):10. doi: 10.1167/tvst.7.2.10

2. Graham B. Kaggle diabetic retinopathy detection competition report. University of Warwick 2015;22.

3. Carpenter J, Bithell J. Bootstrap confidence intervals: when, which, what? A practical guide for medical statisticians. Stat Med 2000;19(9):1141–1164.

4. Unal I. Defining an Optimal Cut-Point Value in ROC Analysis: An Alternative Approach. Comput Math Methods Med 2017;2017:3762651. doi: 10.1155/2017/3762651

5. Simonyan K, Vedaldi A, Zisserman A. Deep Inside Convolutional Networks: Visualising Image Classification Models and Saliency Maps. arXiv preprint arXiv:13126034 2013; doi: 10.48550/arXiv.1312.6034

6. Choi N, Kim Z, Song BH, Park W, Chung MJ, Cho BH, et al. Prediction of risk factors for pharyngo-cutaneous fistula after total laryngectomy using artificial intelligence. Oral Oncol 2021 Aug;119:105357. doi: 10.1016/j.oraloncology.2021.105357

7. Van Rossum G, Drake FL. Python 3 Reference Manual. 2009. ISBN:1-4414-1269-7

8. Charles RH, K. Jarrod Millman, Stfan J van der W, Ralf G, Pauli V, David C, et al. Array programming with NumPy. Nature 2020 Sep;585(7825):357--362. doi: 10.1038/s41586-020-2649-2

9. McKinney W, et al. Data structures for statistical computing in python. 2010.

10. Virtanen P, Gommers R, Oliphant TE, Haberland M, Reddy T, Cournapeau D, et al. SciPy 1.0: Fundamental Algorithms for Scientific Computing in Python. Nature Methods 2020;17:261--272. doi: 10.1038/s41592-019-0686-2

11. Martin~Abadi, Ashish~Agarwal, Paul~Barham, Eugene~Brevdo, Zhifeng~Chen, Craig~Citro, et al. TensorFlow: Large-Scale Machine Learning on Heterogeneous Systems. 2015.

12. Chollet, Francois and others. Keras. GitHub 2015; Available from: https://github.com/fchollet/keras

13. Bradski G. The OpenCV Library. Dr Dobb’s Journal of Software Tools 2000; Available from: https://github.com/opencv/opencv

14. Pedregosa F, Varoquaux G, Gramfort A, Michel V, Thirion B, Grisel O, et al. Scikit-learn: Machine Learning in Python. Journal of Machine Learning Research 2011;12:2825--2830.

15. Robin X, Turck N, Hainard A, Tiberti N, Lisacek F, Sanchez J-C, et al. pROC: an open-source package for R and S+ to analyze and compare ROC curves. BMC Bioinformatics 2011 Mar 17;12(1):77. doi: 10.1186/1471-2105-12-77
